# Predicting diabetes clinical outcomes using longitudinal risk factor trajectories

**DOI:** 10.1186/s12911-019-1009-3

**Published:** 2020-01-08

**Authors:** Gyorgy J. Simon, Kevin A. Peterson, M. Regina Castro, Michael S. Steinbach, Vipin Kumar, Pedro J. Caraballo

**Affiliations:** 1 Department of Medicine, University of Minnesota, Minneapolis, USA; 2 Institute for Health Informatics, University of Minnesota, Minneapolis, USA; 3 Department of Family Medicine, University of Minnesota, Minneapolis, USA; 4 Division of Endocrinology, Mayo Clinic, Rochester, USA; 5 Department of Computer Science and Engineering, University of Minnesota, Minneapolis, USA; 6 Department of Internal Medicine, Mayo Clinic, Rochester, USA

**Keywords:** Diabetes, Diabetes trajectories, Risk assessment, Prediabetes

## Abstract

**Background:**

The ubiquity of electronic health records (EHR) offers an opportunity to observe trajectories of laboratory results and vital signs over long periods of time. This study assessed the value of risk factor trajectories available in the electronic health record to predict incident type 2 diabetes.

**Study design and methods:**

Analysis was based on a large 13-year retrospective cohort of 71,545 adult, non-diabetic patients with baseline in 2005 and median follow-up time of 8 years. The trajectories of fasting plasma glucose, lipids, BMI and blood pressure were computed over three time frames (2000–2001, 2002–2003, 2004) before baseline. A novel method, Cumulative Exposure (CE), was developed and evaluated using Cox proportional hazards regression to assess risk of incident type 2 diabetes. We used the Framingham Diabetes Risk Scoring (FDRS) Model as control.

**Results:**

The new model outperformed the FDRS Model (.802 vs .660; *p*-values <2e-16). Cumulative exposure measured over different periods showed that even short episodes of hyperglycemia increase the risk of developing diabetes. Returning to normoglycemia moderates the risk, but does not fully eliminate it. The longer an individual maintains glycemic control after a hyperglycemic episode, the lower the subsequent risk of diabetes.

**Conclusion:**

Incorporating risk factor trajectories substantially increases the ability of clinical decision support risk models to predict onset of type 2 diabetes and provides information about how risk changes over time.

## Background

The early identification of individual risk for developing type 2 diabetes is essential for effective targeting of preventive measures. Early intervention through lifestyle change and/or metformin therapy have shown robust results in preventing or postponing the onset of diabetes [1, 2]. More precise identification of individual risk allows limited resources to be balanced against individual needs.

Diabetes risk scores, also known as diabetes indices or risk equations, are currently used to estimate individual risk for developing diabetes [3–9]. Besides estimating individual risk, these risk scores can also deepen our understanding of how diabetes develops, and inform us of interactions between a specific risk factor and subsequent development of complications. Many risk scores exist with the sole purpose of risk estimation [6, 7] and numerous diabetes models have been developed for the purpose of biomarker discovery, [10, 11] but very few, if any, models are able to simultaneously address both goals.

The Framingham diabetes score is a widely used model for estimating diabetes risk [12]. In this score, weights are assigned to seven risk factors, and the weights of the risk factors that a patient presents with are summed. The Framingham score is a paper-and-pencil score, [13] which is easy to compute during a patient visit. The ease of computation, however, trades accuracy for simplicity, and hides the heterogeneity and the wide array of clinical risk factors [14] associated with diabetes. In response, a stream of increasingly accurate but increasingly complex risk models followed (see [5, 6, 9, 15] for systematic reviews), often relying on measurements related to nutrition, caloric intake and lifestyle, that are not commonly recorded in routine clinical practice. These scores (or rather equations) are highly multivariate, and are no longer computable with paper and pencil. The adoption of electronic health records (EHR) systems can, in theory, alleviate the problems stemming from running complex predictive models; however, the reliance of these diabetes scores on data elements not commonly available in the EHR system renders these models impractical. More importantly, the pursuit of increasingly marginal improvements in predictive accuracy and the lack of temporal frames limit our understanding of the disease and its progression.

We have previously shown that the order in which patients develop comorbidities is predictive of the risk of diabetes, even after adjusting for the severity of the comorbidities [16]. In another study, Hulsegge et al. compared trajectories of laboratory results and vital signs between patients who developed diabetes and those who did not, over 21 years, taking a snapshot every 5 years. They showed that laboratory results can be different as many as 15–20 years before the diagnosis of diabetes, but they did not associate trajectories with risk of diabetes [17]. None of the diabetes risk scores take patient trajectory into account.

In this paper, we develop a novel methodology, Cumulative Exposure, to associate trajectories of lab results observed at a finer granularity with incidence of type 2 diabetes. The model embraces both goals of risk score development: it offers state-of-the-art prediction accuracy using only data elements that we extracted from our EHR system and it simultaneously allows us to generate new hypothesis about the temporal aspect of diabetes.

## Methods

### Study setting

Mayo Clinic provides primary care to residents of Olmsted County, Minnesota, and it has an integrated electronic health record system including diagnoses, medications, laboratory results and clinical notes. These records are part of the Rochester Epidemiology Project (REP), a comprehensive research data repository over several decades, approved for medical research. The resources available for the REP have been described elsewhere [18]. The primary care clinics at Mayo Clinic provide routine health care similar to any primary care clinic elsewhere. The study was approved by Mayo Clinic IRB.

### Study design

We used a retrospective cohort of de-identified data from 71,454 primary care patients at Mayo Clinic, Rochester, MN with research consent. The cohort consists of patients aged ≥18 at baseline on Jan. 1st, 2005, having at least one visit before and after baseline. These patients were followed until 2015 (median follow-up time is 8 years). We extracted diagnoses (ICD-9), laboratory results, vital signs, and medications longitudinally for three non-overlapping time periods: 2000–2001, 2002–2003 and the year of 2004. Patients with pre-existing diagnosis of diabetes at baseline (5891 patients), without fasting plasma glucose (FPG) measurements during any of the three time periods (32,852) and those with suspected diabetes (indicated by insulin or oral antidiabetic medication use or a single FPG > 125 mg/dl; 2427 patients) at any time before baseline were excluded. The final cohort consists of 30,284 patients. Table 1 contains a description of the cohort.

**Table 1:** Description of the cohort. For lab results and vitals, the median and interquartile range and for medication usage and progression to diabetes the number and percentage of patients are reported

Variable	Median	Interquartile Range
Age [years]	51	41, 62
Male [%]	38.4	
LDL [mg/dL]	111	91, 32
TG [mg/dL]	114	81, 161
HDL [mg/dL]	52	43, 64
SBP [Hg mm]	122	110, 132
DBP [Hg mm]	73	66, 80
FPG [mg/dL]	92	87, 99
Follow-up [years]	9.5	8.0, 9.8
	Number of patients	Percent
Antihypertensive medication	6571	21.7
Antilipemic medication	5395	17.8
Progressed to DM	2972	9.8

### Predictors

The predictor variables include age, gender, ICD-9 diagnoses categorized into four diabetes risk factors (hypertension, dyslipidemia, impaired fasting glucose, obesity) and medication use for the above categories rolled up to National Drug File Reference Terminology NDF-RT pharmaceutical subclasses at baseline, vital signs (BMI, systolic and diastolic blood pressure; SBP and DBP, respectively), and laboratory results (LDL, HDL, triglycerides, and fasting plasma glucose; FPG). Glucose values were used if they were fasting glucose value obtained during routine clinical care in the ambulatory setting. Glucose values done in the emergency department and hospital setting were excluded. Point-of-care glucose measurements, which usually use capillary whole-blood, were also excluded. All the laboratory tests were done by Mayo Clinic Laboratories which are fully certified by the College of American Pathology and the Clinical Laboratory Improvement Amendments. These data yield three sets of predictor variables. The first set is *baseline*, and it contains the latest measurements before baseline. The second set is *extreme measurements*, which contains the most extreme (minimum for HDL, maximum for the others) result over the 5-year period of 2000–2004. The third set is the proposed *cumulative exposure.* Through linear interpolation, a segment-wise linear curve of the lab results and vital signs were obtained, and the area under the curve was computed for three non-overlapping time periods: 2000–2001, 2002–2003, and 2004. If the curve could not be estimated via linear *inter*polation for a time period (e.g. there was no result before 2000 for the 2000–2001 time period), the cumulative exposure variable for that time period was marked missing. To complete the curve between the last measurement and Jan 1st, 2005, the last measurement was carried forward (the measurement was assumed to stay constant). The cumulative exposure can be interpreted as our best estimate of the average of the daily lab values of the patient for each time period.

### Outcomes

The study endpoint was incident type 2 diabetes mellitus as defined by a first ICD-9 diagnosis code or a fasting glucose measurement in excess of 125 mg/dl.

### Statistical modeling

Cox proportional hazards regression models were constructed with type 2 diabetes mellitus (T2DM) as the dependent variable using age, gender, and some of the above sets of clinical predictor variables. Specifically, four models were constructed:

1. **Baseline** using demographic information (age, gender) and the *baseline* predictors (latest lab results and vital signs before baseline);
2. **Cumulative Exposure (CE)** using demographics, *baseline* and the *cumulative exposure* variables;
3. **Extreme values (EV)** using demographics, *baseline* and the *extreme measurements* (most extreme lab results and vitals over 2000–2004); and
4. **Extreme plus Cumulative (EV + CE)** which uses all variable sets (demographics, *most recent*, *extreme measurement*, and *cumulative exposure*).

Laboratory results and vital signs completely missing throughout the years 2000–2005 were handled through mean imputation with the addition of missingness indicator variables. When results were missing for one of the three time periods, carry-forward imputation was used. Patients with missing fasting glucose measurements were discarded. Backwards elimination was used for variable selection.

The four models were compared to the Framingham Diabetes Risk Scoring Model (FDRSM) [12].

### Model evaluation

Model performance was evaluated using bootstrap estimation with 1000 replications and survival concordance as the evaluation metric measured on the out-of-bag samples. Survival concordance is the probability that for any pair of patients in which one patient remained free of progression to overt diabetes longer than the other, the one who developed diabetes earlier has higher predicted risk. Survival concordance is the C-statistic for censored data. We report the model performances as the median, upper and lower quartiles of the 1000 performance measurements. All models were evaluated on the same 1000 replications, so paired t-test was used for pairwise comparison of model performances.

### Applying CE to study episodic prediabetic populations

We apply the Cumulative Exposure model to study the effect of *episodic* pre-diabetes on incident diabetes. By ‘episodic prediabetes’, we refer to a short (no more than 2–3 years long) episode of prediabetes (FPG between 100 and 125 mg/dl) where the patient returned to normoglycemia without pharmacological intervention. We study two subpopulations that differ in the duration of normoglycemia following the prediabetic episode and two kinds of controls: patients who did not return to normoglycemia (two subpopulations) and patients who did not develop prediabetes. Specifically, we have the following subpopulations:

1. patients who were prediabetic in 2000–2001 and returned to normoglycemia in 2002–2003 (‘pnn’);
2. patients who were prediabetic in 2002–2003 and returned to normoglycemia in 2004 (‘npn’);
3. patients who became prediabetic in 2004 (‘nnp’);
4. patients who were normoglycemic in 2001–2002 and developed prediabetes in 2002–2003 (‘npp’);
5. patients who did not developed prediabetes before 2005 (‘nnn’).

We fit the Baseline, the Cumulative Exposure, and the Extreme Value models to the entire population as described above. Missing value imputation was applied to the entire population before the subpopulations were created. We used these models to estimate the risk of developing overt diabetes in these specific subpopulations. We defined our risk as the per-patient expected number of diabetes incidents in each subpopulation during the 10 follow-up years (2005–2015) and we defined the error as the (signed) Martingale residual (difference between the per-patient estimated and observed number of diabetes incidents). We wish to know how diabetes risk varies across the subpopulations and how well the two models can estimate them.

### Sensitivity Analysis

We carried out a sensitivity analysis in patients with at least one FPG measurement in all three time periods (2000–2001; 2002–2003; and 2004) and at least five during follow-up to ascertain that our conclusions are not unduly impacted by the intermittent nature of the patient visits.

## Results

### Baseline cohort characteristics

Table 1 shows the clinical characteristics of the cohort at baseline, 2005.

### Performance of the predictive models

**Fig. 1:**
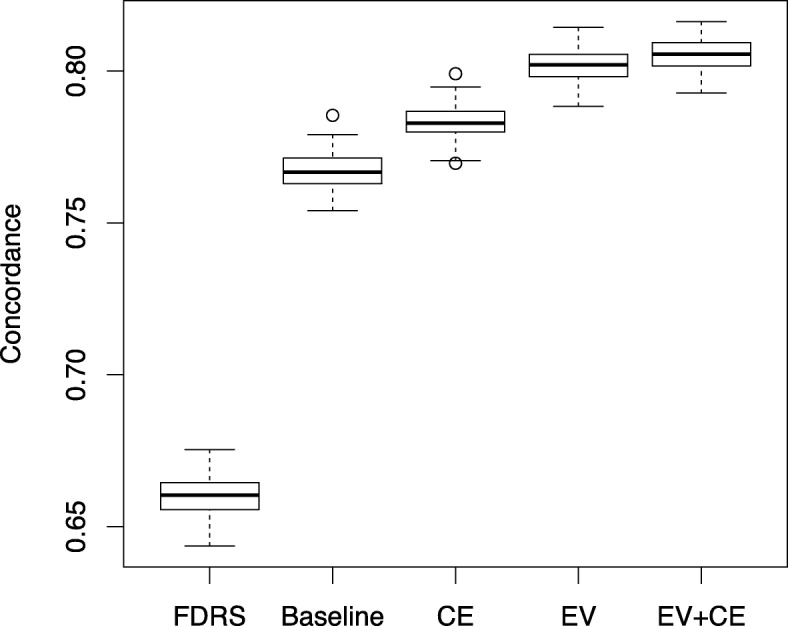
Performance comparison of the four regression models and the Framingham score. FDRS: Framingham Diabtes Risk Score, CE: Cumulative Exposure, EV: Extreme Value, EV + CE: Extreme Plus Cumulative Exposure. Performance is measured through survival concordance using bootstrap estimation with 1000 replications. The performance difference between any two models is statistically significant at .05 level

The performance of the new models, Baseline, Cumulative Exposure, Extreme Values and Extreme plus Cumulative Models, each outperformed the FDRS Model, with concordance of 0.767, 0.783, 0.802, 0.805 and 0.660 respectively, all *p*-values <2e-16 (Fig. 1). Among the four new models, only Baseline lacks the ability to take the patient’s past trajectory into account and accordingly has a substantially lower performance than the other models.

Table 2 shows the coefficients of the statistically significant laboratory results and vital signs after backwards elimination in each model. Each row within a model corresponds to a variable set and timeframe (baseline, extreme measurements, cumulative exposure over the three timeframes labeled as 2000-2001, 2002-2003 and 2004) and each column corresponds to a laboratory result or vital sign. Consider, for example, the effect of FPG (column ‘fasting’) in the Cumulative Exposure model. The Cumulative Exposure model is the second group from the top in Table 2 and has four rows (timeframes): 2000-2001, 2002-2003, 2004 and baseline. A unit increase in the cumulative exposure of FPG, which is essentially the estimated daily average FPG level, in the timeframe of 2000-2001 independently increases the relative hazard of diabetes by exp(.034)=1.04. Additionally, a unit increase in 2002-2003 further increases the relative hazard (independently of other timeframes) by exp(.035)=1.04 and the baseline measurement increases it further by exp(.041). The cumulative exposure to FPG in 2004 was not significant (because the most recent FPG is mostly the only measurement from 2004). The other lab results and vitals can be interpreted analogously.

**Table 2:** Coefficients of the four models organized by timeframe. 2000–2001 refers to the cumulative exposure between 01/01/2000 and 12/31/2001; 2001–2002 refers to the cumulative exposure between 01/01/2002 and 12/31/2003; 2004 is the cumulative exposure in 2004; ‘Baseline’ refers to the latest observations before baseline; and ‘5-year extreme’ refers to the most extreme (minimum for HDL and maximum for others) observations between 01/01/2000 and 12/31/2004

	fasting	bmi	sbp	dbp	ldl	hdl	trigl
Baseline							
Most recent	0.064	0.009	0.011			−0.021	0.002
Cumulative Exposure							
2000–2001	0.034	0.010					0.001
2002–2003	0.035				−0.002		
2004		0.011					
Most recent	0.041	0.005	0.009			−0.017	0.001
Extreme Values							
Most recent	0.018	0.011	0.009			−0.018	0.001
5-year extreme	0.072	−0.004		0.004	−0.001		0.001
Extreme Plus Cumulative							
2000–2001	0.008	0.016		−0.009			
2002–2003	0.022	0.018					
2004							
Most recent	0.015	0.013	0.008			−0.015	0.001
5-year extreme	0.062	−0.009		0.009	−0.001		0.001

### Using the cumulative exposure model to study episodic pre-diabetic subpopulations

Table 3 presents a comparison of the five subpopulations in terms of their median lab results, vitals, age, follow-up time, and percentage of medication use. None of the patients in the cohort used anti-diabetic medications.

**Table 3:** Comparison of the 5 subpopulations. The label of the subpopulation is derived from the diabetes status of the patients in the three time periods: 2000–2001, 2002–2003, and 2004. For example, ‘pnn’ patients were pre-diabetic in 2000–2001, normal in 2002–2003 and normal in 2004; ‘npn’ patients were normal in 2000–2001, prediabetic in 2002–2003 and normal in 2004. The other subpopulation labels can be interpreted analogously

	All	pnn	npn	nnp	npp	nnn
N	30,284	2181	1193	1065	889	14,387
Age [median; years]	51	55	56	54	55	49
Male [%]	38	42	42	37	48	32
LDL [median; mg/dL]	111	111	109	113	113	111
TG [median; mg/dL]	114	120	118	129	135	106
HDL [median; mg/dL]	53	54	52	53	50	56
SBP [median; Hg mm]	122	124	124	124	125	120
DBP [median; Hg mm]	73	74	74	74	76	72
FPG [median; mg/dL]	92	93	93	104	105	89
BMI [median; kg/m^2^]	27	28	28	29	29	26
Antihypertensive medication [%]	21.7	27.0	32.4	29.9	29.8	17.6
Antihyperlipidemi medication [%]	17.8	22.2	27.8	22.5	23.3	15.2
Fullow-up [median; years]	9.51	9.55	9.55	9.56	9.43	9.59
Diabetes outcome [%]	9.8	8.9	12.2	14.1	19.9	4.4

Table 4 summarizes the estimates from the Most Recent, the Cumulative Exposure, and the Extreme Value models for five subpopulations. The results from the Extreme Plus Cumulative model are very similar to the Extreme Value model, so we omitted them from Table 3. For each model, the estimated risk (Pred) and the estimation error (Error) are displayed. We will refer to each group by their row number.

**Table 4:** Estimating diabetes risk in subpopulations that developed pre-diabetes at different time points. Some groups returned to normoglycemia thereafter. The table shows the number of patients (N), mean predicted diabetes risk as the expected number of incidents in 10 years (Pred) and the estimation error (Error) by the Baseline, Cumulative Exposure, and the Extreme Value models

Subpopulation	N	Baseline		Cumulative		Extreme	
		Pred	Error	Pred	Error	Pred	Error
PreDM in 2000–2001, normal from 2003 onwards	2181	.135	.006	.145	.001	.181	−.018
PreDM in 2002–2003, normal before and after	1193	.157	.026	.175	.019	.262	−.034
Normal before 2004, PreDM in 2004	1065	.332	−.057	.235	.001	.282	−.234
Normal before 2002–2003, PreDM since then	889	.411	−.029	.384	−.008	.402	−.019
Normal throughout 2000–2004	14,387	.092	−.016	.080	−.007	.069	−.001

#### Risk of diabetes in the five subpopulations

Patients who returned to normoglycemia after an episode of hyperglycemia (groups 1 and 2) had lower risk of progression to overt diabetes than patients who did not return to normoglycemia (groups 3 and 4): the adjusted risks in groups 1 and 2 were .087 and .103, as compared to groups 3 and 4, where it was .139 and .207 as estimated by the Cumulative Exposure model. Patients who returned to normoglycemia (groups 1 and 2) had higher adjusted risk of developing overt diabetes than patients who did not develop prediabetes (group 5): the adjusted risk of diabetes was .087 and .103 vs .051 by the Cumulative Exposure model. The risk estimates from the Baseline and the Extreme Value model show similar trends but with higher estimation errors.

In patients who returned to normoglycemia after an episode of documented fasting hyperglycemia, and patients who had an episode of hyperglycemia earlier (and hence remained normoglycemic longer) had a lower risk of progression to diabetes. The adjusted risk by the Cumulative Exposure model for patients who had their prediabetic episode in 2000–2001 was .087 vs .103 for those who had it in 2002–2003.

#### Accuracy of the estimation

The estimation error for the Cumulative Exposure model was 1.5 to 50 times lower than for the Baseline model: it was highest in group 3 with .057 vs .001 and lowest in group 2 with .026 vs .019. In the predominant group (group 5 with 14,387 patients), the CE model had less than half the error of the Baseline model (.007 vs .016). In all groups except group 3, the estimation error of CE was lower than 1%. In contrast, the Baseline model had estimation errors as high as 5.7% and had an estimation error less than 1% only in one subpopulation (group 1). The Extreme Value model had almost perfect estimate in group 5 (patients who did not develop prediabetes) with an estimation error less than one tenth of a percent, but it had higher estimation error than the Cumulative Exposure model in all other groups, and it even had higher estimation error than the Baseline model in the first three groups.

### Sensitivity analysis

Results from the sensitivity analysis show similar tendencies as Table 4.

## Discussion

### Predictive performance of the models

Our results showed that it is possible to build diabetes risk models with state of the art predictive performance using variables that are commonly available in the electronic health records. Among the four models we constructed, the Baseline model, which is built using diagnoses, medication prescriptions, lab results (lipids and FPG) and vitals (blood pressure and BMI) at baseline, and does not even take trajectories into account, achieved a survival concordance of .767 (±.006). This performance represents a 14% improvement over the performance of the Framingham score (.660 ± .006) and is highly comparable to the performance of state-of-the-art risk models published in a large validation study [7]; thus, the Baseline model can be considered a state-of-the-art model in its own right.

Taking historic information about laboratory results and vital sign into account significantly improves predictive accuracy. The simplest way to incorporate history is to compute the most extreme measurement during the period of 2000–2004. Adding these predictors to the Baseline model results in the Extreme Value model, which has almost 5% higher concordance than the Baseline model (.802 vs .767; *p*-value < 2.2e-16). Having one historic measurement in 2000–2004 and the most recent measurement for most patients forms a trajectory, albeit a very crude one. The results from the Extreme Value model show that incorporating *any* trajectory information is very beneficial; even this crude representation of a trajectory brought almost half as much improvement as adding all the predictors to the Framingham score that the Baseline model has.

Finally, the cumulative exposure variables refined the notion of trajectories, further improving the performance to .805 (±.005). This improvement is important because it represents a substantial difference in certain subpopulations. The key difference between the Extreme Model and the Cumulative Exposure model is granularity, which gives us two pieces of information: (i) the time frame in which the extreme value occurred, and (ii) whether or not the patient was normal in other time frames. The cumulative exposure model opens up a temporal dimension, allowing us to directly model situations where patients can have intermittent abnormal laboratory results, and are brought back under control through (say) lifestyle changes. The Cumulative Exposure achieved higher predictive ability to assess the risk of diabetes in patients who had prediabetes at some point in the past than the Baseline or the Extreme Values model.

### Importance of incorporating trajectories

Not only does incorporating trajectories through the Cumulative Exposure variables improve predictive performance, it also improves our understanding of diabetes. While many of the metabolic risk factors of diabetes are well known, [14, 19] their temporal behavior is not.

We have demonstrated through the use of the cumulative exposure model that even episodic (short-term; no more than 2–3 years of) prediabetes increases the risk of developing overt diabetes, and that returning to normoglycemia mitigates this risk, but does not fully eliminate it. We could not find any evidence in the literature indicating whether or not returning to normoglycemia eliminates the increase in risk possibly caused by previous prediabetes, it is well understood that prediabetic patients face an increased risk of developing type 2 diabetes, and it is also known that *sustained* successful intervention either via lifestyle change or pharmacological intervention can delay the onset of diabetes by 4–5 years [1, 2].

Moreover, our results also suggest that the longer a patient remains normoglycemic after an episode of hyperglycemia, the lower the risk of developing diabetes. Our results suggest that temporarily returning to normoglycemia between two episodes of hyperglycemia has a positive effect on mitigating the risk of developing diabetes.

### Obesity trajectories

While we did not perform a subpopulation analysis specifically for obese patients, the coefficients of the Cumulative Exposure model suggest that an analogous relationship exists between BMI and obesity. Becoming obese even for a short period of time increases the patient’s risk of developing overt type 2 diabetes and losing weight thereafter mitigates this risk. Similar to prediabetes, the effect of previous short-term obesity is attenuated over time: the longer the patient has been non-obese, the lower the effect of any previous incidence of obesity. After 5 years, the effect of previous obesity appears to lose any significant effect. This observation requires a cautionary statement. When exactly the effect becomes insignificant depends on the sample size, thus the 5-year period we observed in our sample may be a statistical artifact, but the attenuation in the effect size is not. In other words, in a larger cohort, 2000–2001 BMI could have been statistically significant, but we expect its effect size to be smaller than the effect size in 2002–2003.

### Metabolic memory

In the context of progression from diabetes to its complications, the concept of metabolic memory of glucose control has been proposed. Several studies have shown that better early glycemic control has enduring effect that persists over time [20]. For example, in the Diabetes Control and Complications Trial (DCCT), patients with type 1 diabetes were randomized to intensive or standard insulin regimens to control their blood sugars [21]. Because the group in the intensive arm achieved profound reductions in the rate of microvascular complications, the trial was stopped early and all patients were switched to intensive therapy. In a follow up trial with this same population (EDIC trial) it was found that those initially assigned to the intensive arm continued to have lower incidence of complications despite the fact that both groups had subsequently achieved similar glycemic control for several years after switching to the intensive therapy [22]. In other words, initial better glucose control has sustained long-term benefits.

Our study presents a complementary but compatible viewpoint. We found that even short-term loss of control can result in long-term disadvantages. Exposure to hyperglycemia also has “memory”: elevated FPG in the past continues to increase risk of diabetes in the future despite having achieved similar control (returning to normoglycemia). However, our study also suggests that this memory fades over time. Returning to normoglycemia attenuates the negative effect of prior exposure. Our findings are compatible with previous findings in the sense that among patients with similar control (normoglycemic at baseline), achieving better control (return to normoglycemia earlier) has future benefits.

Although the UKPDS blood pressure control trial failed to demonstrate “memory” for blood pressure, we found that cumulative exposure to elevated blood pressure was significant for the most recent timeframe [23]. This could be due to loss of power, since patients with missing blood pressure measurement during 2000–2004 were included, while patients with similarly missing glucose were excluded.

### Limitations

Our study cohort was defined so that patients have multiple FPG measurements; however, other laboratory results and vital signs could be missing. Specifically, there are 2200 patients who have no blood pressure measurements and 1600 patients who have no lipid measurements during the entire period of 2000–2004. The lack of statistical significance of lipid trajectories may be due to the lower statistical power of these variables. These results are only applicable to health care provided in the ambulatory care setting, specifically, primary care, and using fasting plasma glucose measurements.

This is a single center study, with limited racial variability; therefore, the effect of race could not be incorporated. Social history and family history was available only for a limited number of patients. The study was based on EHR data. As such, non-pharmacological interventions, such as lifestyle changes, were not consistently documented.

## Conclusion

We have demonstrated that laboratory results and vital sign trajectories that can be extracted from EHR data provide better risk estimates than current models using baseline measurements. The metabolic memory of exposure to even mildly elevated glucose levels exists, but fades over time when glucose is under control. Incorporating these data into risk estimates provides better identification of individual risk, and allows for allocation of resources to be more precisely balanced against individual need.

## Data Availability

The data analyzed in this manuscript is patients’ health information coming from the Rochester Epidemiology Project (REP) at Mayo Clinic. This is patients’ private information and we are unable to share it publicly.

## Abbreviations

BMI: Body mass index
CE: Continuous Exposure (model)
DBP: Diastolic Blood Pressure
EHR: Electronic Health Records
EV: Extreme Value (model)
FDRS: Framingham Diabetes Risk Score
FPG: Fasting Plasma Glucose
HDL: High Density Lipoprotein
LDL: Low Density Lipoprotein
REP: Rochester Epidemiology Project
SBP: Systolic Blood Pressure
T2DM: Type-2 Diabetes Mellitus
TG: Triglycerides
